# De novo biosynthesis of β-arbutin in *Corynebacterium glutamicum* via pathway engineering and process optimization

**DOI:** 10.1186/s13068-024-02540-2

**Published:** 2024-06-25

**Authors:** Bin Zhang, Kexin Gou, Kexin Xu, Zhimin Li, Xiaoyan Guo, Xiaoyu Wu

**Affiliations:** 1 College of Bioscience and Bioengineering, Jiangxi Agricultural University, Nanchang, 330045 Jiangxi China; 2 Jiangxi Engineering Laboratory for the Development and Utilization of Agricultural Microbial Resources, Jiangxi Agricultural University, Nanchang, 330045 Jiangxi China

**Keywords:** *Corynebacterium glutamicum*, β-Arbutin, Shikimate pathway, Metabolic engineering, Fermentation optimization

## Abstract

**Background:**

β-Arbutin, a hydroquinone glucoside found in pears, bearberry leaves, and various plants, exhibits antioxidant, anti-inflammatory, antimicrobial, and anticancer effects. β-Arbutin has wide applications in the pharmaceutical and cosmetic industries. However, the limited availability of high-performance strains limits the biobased production of β-arbutin.

**Results:**

This study established the β-arbutin biosynthetic pathway in *C. glutamicum* ATCC13032 by introducing codon-optimized *ubiC*, *MNX1*, and *AS*. Additionally, the production titer of β-arbutin was increased by further inactivation of *csm* and *trpE* to impede the competitive metabolic pathway. Further modification of the upstream metabolic pathway and supplementation of UDP-glucose resulted in the final engineered strain, *C. glutamicum* AR11, which achieved a β-arbutin production titer of 7.94 g/L in the optimized fermentation medium.

**Conclusions:**

This study represents the first successful instance of de novo β-arbutin production in *C. glutamicum*, offering a chassis cell for β-arbutin biosynthesis.

**Supplementary Information:**

The online version contains supplementary material available at 10.1186/s13068-024-02540-2.

## Introduction

Arbutin, a hydroquinone (HQ) glucoside found in various plants, including pears, wheat, coffee, and tea [1–3], is extensively used in skin-whitening cosmetics because of its ability to inhibit melanin formation [4]. Additionally, studies have described arbutin’s antioxidative properties [5], anti-inflammatory effects [6], antimicrobial activity [7], and anticancer attributes [8], broadening its scope for various applications [9]. Efforts to enhance arbutin production have become a research priority driven by the increasing demand for this compound. The current methods for obtaining arbutin include plant extraction, chemical synthesis, biological catalysis, and microbial fermentation. Chemical synthesis and biotransformation are the primary approaches for industrial production of arbutin [10, 11]. Numerous glycosyltransferases (GTs), including sucrose phosphorylase [12] and amylosucrase [13], have been demonstrated to catalyze the synthesis of α-arbutin from HQ and various glycosyl donors [10]. The most substantial results regarding α-arbutin yield have been achieved utilizing recombinant *Escherichia coli* as the catalyst, with levels reaching 102–108 g/L [14]. Despite the high yield of α-arbutin achieved through biotransformation, challenges associated with enzyme purification and adding the toxic compound HQ have compounded the difficulties in arbutin production [15]. The toxicity of HQ surpasses that of phenol, further complicating its chemical synthesis and biotransformation processes.

The diverse chemical capabilities of various metabolic pathways make it possible to create a robustly engineered microorganism to produce β-arbutin using heterologous biosynthetic routes [16]. Engineered microorganisms for chemical biosynthesis offer several advantages, including reliance on cost-effective carbon sources, ease of cultivation with rapid growth, access to genetic technologies, and well-established metabolic networks [17, 18]. The generation of β-arbutin relies on chorismate, a vital metabolite in the shikimate pathway. The initial phase of β-arbutin synthesis involves the action of chorismate pyruvate lyase, encoded by *ubiC* in *E. coli*, which facilitates the conversion from chorismate to p-hydroxybenzoic acid (pHBA) [19]. Subsequently, the biosynthesis of HQ from pHBA, the precursor for β-arbutin synthesis, is catalyzed by the flavin adenine dinucleotide (FAD)-dependent enzyme 4-hydroxybenzoate 1-hydroxylase (encoded by *MNX1*), which has been identified in *Candida parapsilosis* CBS604 [20]. Finally, the synthesis of β-arbutin from glucose was made possible by introducing GTs. β-Arbutin synthase (encoded by *AS*) from *Rauvolfia serpentina*, identified as a novel member of Class IV GTs, has been demonstrated to catalyze the synthesis of β-arbutin from HQ using uridine diphosphate glucose as the glycosyl donor [21, 22]. This enzyme has high specificity for HQ (100%) and low activity (< 10%) for HQ analogs [22]. Introducing three heterologous enzymes (UbiC, MNX1, and AS) can potentially redirect the carbon flux from the shikimate pathway to the biosynthesis of β-arbutin. The feasibility of this synthetic pathway has been demonstrated in *E. coli* [23, 24], *Yarrowia lipolytica* [25], and *Pseudomonas chlororaphis* P3 [26], with maximum yields of 43.79 g/L [27]. However, the limited yield hampers the industrial application of this biobased β-arbutin production method. Screening for robust β-arbutin-producing strains as cell factories is a current research focus that aims to improve the microbial fermentation production of β-arbutin further.

*Corynebacterium glutamicum*, a generally recognized as safe (GRAS) strain, manufactures high-value-added compounds [28, 29]. Recently, significant efforts have been directed toward engineering *C. glutamicum* to produce aromatic compounds [30]. Engineered *C. glutamicum* reportedly produces 141 g/L shikimate [31], 36.6 g/L pHBA [32], and 43 g/L *para*-aminobenzoate [33]. These levels represent the highest yields of microbial fermentation, indicating the utility of *C. glutamicum* for producing aromatic compounds. Similarly, β-arbutin is biosynthesized from chorismate, an intermediate metabolite of the shikimate pathway. Thus, *C. glutamicum* appears to have considerable potential for β-arbutin production.

Our laboratory has been committed to the metabolic engineering of *C. glutamicum* for a long time and has made progress in producing many products. To establish a recombinant strain of *C. glutamicum* capable of producing β-arbutin from an inexpensive carbon source, we introduced the *ubiC*, *MNX1*, and *AS* genes into *C. glutamicum* ATCC13032 using two expression plasmids. The fermentation parameters were then optimized. To increase the supply of chorismite and uridine diphosphate (UDP)-glucose, we overexpressed genes encoding key enzymes in the upstream synthetic pathway and blocked the competing metabolic pathways. To our knowledge, this is the first report on the biosynthesis of β-arbutin in *C. glutamicum*.

## Results and discussion

### Tolerance of *C. glutamicum* to HQ and β-arbutin

**Fig. 1:**
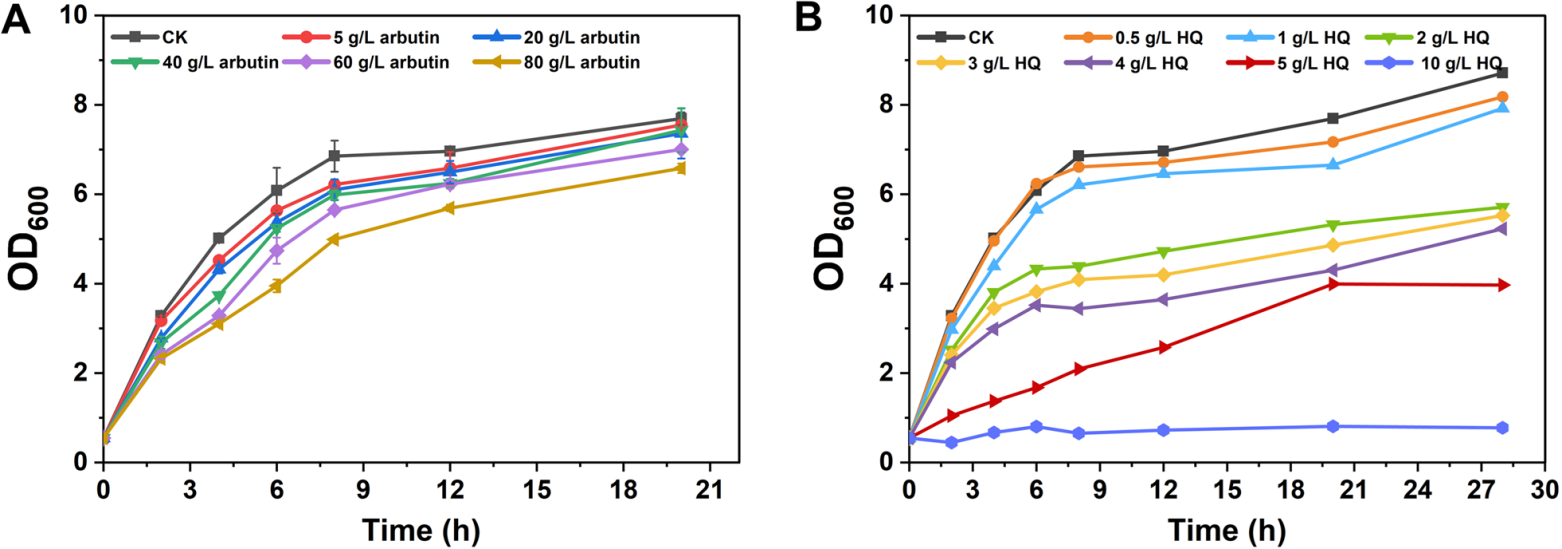
Tolerance experiments of *C. glutamicum*. **A** Growth curves of *C. glutamicum* in 20 h after adding 0, 5, 20, 40, 60, and 80 g/L β-arbutin to LB medium, respectively. **B** Growth curves of *C. glutamicum* in 28 h after adding 0, 0.5, 1, 2, 3, 4, 5, and 10 g/L HQ to LB medium, respectively

As the standard of living improves and the demand for nutritional and cosmetic products continues to rise, the demand is growing for higher yields of β-arbutin. Due to HQ's high toxicity, microbial fermentation is a promising method for producing β-arbutin [14]. This requires the development of safe and robust strains for β-arbutin production. The tolerance of host cells to the target products is often the rate-limiting step in developing classically engineered cells. In this study, to assess the feasibility of β-arbutin synthesis in *C. glutamicum*, we examined the tolerance of *C. glutamicum* to β-arbutin and HQ. The growth of *C. glutamicum* ATCC 13032 was measured after adding various concentrations of β-arbutin. A slight decrease in growth was observed with increasing β-arbutin concentrations. Specifically, when *C. glutamicum* was grown in an LB medium containing 80 g/L of β-arbutin, the OD_600_ was only 14.5% lower than that of the control (Fig. 1A). These results indicate that *C. glutamicum* exhibits a high tolerance to β-arbutin, facilitating the overproduction of β-arbutin. In contrast, *C. glutamicum* tolerates low HQ concentrations (not exceeding 1 g/L). Specifically, when 5 g/L HQ was added to the LB medium, the OD_600_ was 45.6% that of the control. Cell growth was completely inhibited in the LB medium containing 10 g/L HQ (Fig. 1B). These growth inhibition experiments suggest that *C. glutamicum* has significant potential for β-arbutin synthesis without excessive accumulation of the intermediate metabolite HQ.

### De novo biosynthesis of β-arbutin in *C. glutamicum*

**Fig. 2:**
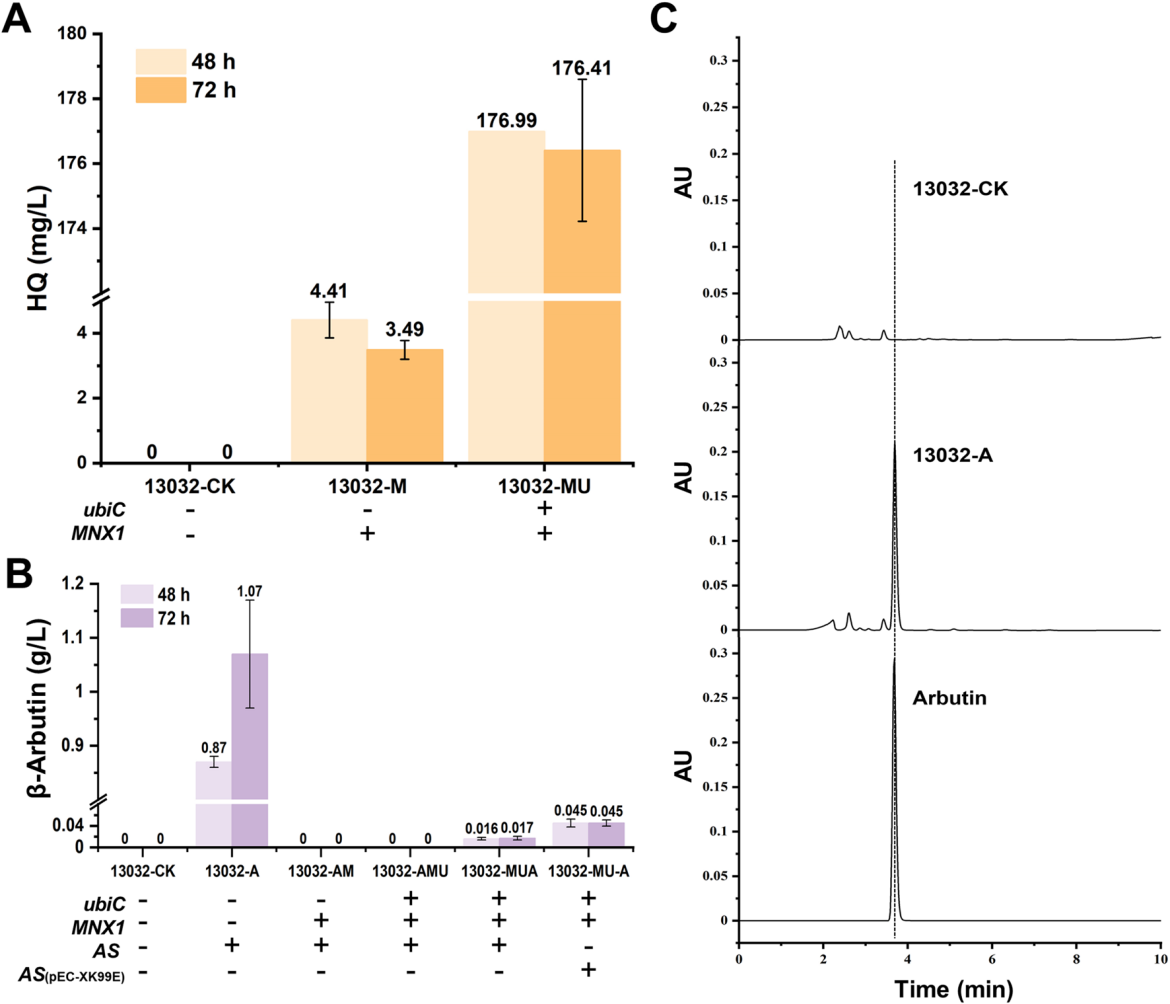
Feeding experiments of pHBA, HQ, and de novo biosynthesis of β-arbutin from glucose. **A** The yield of HQ in recombinant strains. **B** The yield of β-arbutin in recombinant strains. **C** Comparison of HPLC analysis of the β-arbutin standard and the products in fermentation supernatant of recombinant strain AR01, control strain 13032-CK

To achieve this de novo synthesis of β-arbutin in *C. glutamicum*, three codon-optimized exogenous genes—*ubiC* from *E. coli*, *MNX1* from *C. parapsilosis* CBS604, and *AS* from *Rauvolfia serpentina*—were employed for the assembly of the β-arbutin synthetic pathway. These genes have been successfully introduced and shown to function in *E. coli* [23], *Y. lipolytica* [25], and *P. chlororaphis* P3 [26]. Initially, single-gene expression experiments were performed using the plasmid pXMJ19 to generate the engineered strains 13032-A, 13032-M, 13032-U, 13032-AM, 13032-MU, and 13032-AMU. The purpose was to assess whether these genes could adequately express and function in *C. glutamicum*. As a control, empty plasmid pXMJ19 was transfected into *C. glutamicum* ATCC 13032 to obtain strain 13032-CK. The production capabilities of the recombinant strains were evaluated in liquid LB medium supplemented with the corresponding precursors. Notably, the engineered strain 13032-MU, which involved the tandem expression of *MNX1* and *ubiC*, and strain 13032-M supplemented with 1 g/L pHBA, produced 176 and 3.49 mg/L HQ, respectively, after 72 h of cultivation (Fig. 2A and Table 1). In contrast, HQ was not detected in the fermentation supernatant of the control strain, 13032-CK. The presence of HQ in the fermentation supernatant of strain 13032-MU indicates the effectiveness of codon-optimized *MNX1* and *ubiC* in *C. glutamicum* ATCC 13032. No pHBA accumulation was observed in the fermentation broth of strain 13032-U (Table 1). The absence of detectable pHBA levels when *ubiC* was expressed may be attributed to its concentration falling below the detection threshold. In addition, pHBA may not be transported outside the cell after intracellular synthesis. Furthermore, no β-arbutin was detected in the fermentation supernatant of strain 13032-AMU when *MNX1*, *ubiC*, and *AS* were tandemly expressed (Table 1). However, strain 13032-A, which harbored plasmid pXMJ19-AS and was cultured in the presence of HQ, produced 1.07 g/L β-arbutin, indicating the effectiveness of codon-optimized *AS* in *C. glutamicum* (Fig. 2B and Table 1). HPLC analysis revealed a characteristic peak at 3.8 min in the chromatogram compared to the control, with a retention time matching that of the β-arbutin standard (Fig. 2C). The expression of *MNX1* and *AS* was influenced by the position that led to the unsuccessful synthesis of β-arbutin. To test this hypothesis, we positioned the *AS* gene downstream of *MNX1* and *ubiC* to construct the strain 13032-MUA containing the expression plasmid pXMJ19-MUA. Additionally, strain 13032-MU-A containing the pXMJ19-MU and pEC-XK99E-AS plasmids was constructed. In line with our hypothesis, strains 13032-MUA and 13032-MU-A accumulated 17.01 and 45.18 mg/L of β-arbutin during 72 h of cultivation (Fig. 2B and Table 1). The observed accumulation of β-arbutin suggests successfully incorporating the β-arbutin synthesis pathway into *C. glutamicum*, as has been demonstrated for *E. coli* [23], *Y. lipolytica* [25], and *P. chlororaphis* P3 [26]. Nevertheless, the notable difference in β-arbutin yield compared to that in the feeding experiment may be due to an insufficient supply of precursors in *C. glutamicum*. Optimization of pathway engineering to improve the fermentation performance of recombinant strains is required. The engineered strain 13032-MU-A will be renamed AR01 for convenience in future studies.

**Table 1:** Analysis of intermediate metabolites during fermentation process

Strains	Feeding substrate (1 g/L)	β-Arbutin (mg/L)	HQ (mg/L)	pHBA (mg/L)
13032-CK	–	0	0	0
13032-A	HQ	1070.03 ± 201.21	290.11 ± 32.01	0
13032-M	pHBA	0	3.49 ± 0.29	990.12 ± 0.09
13032-U	–	0	0	0
13032-AM	pHBA	0	0	988.57 ± 0.34
13032-MU	–	0	176.41 ± 2.19	0
13032-AMU	–	0	0	0
13032-MUA	–	17.00 ± 0.74	5.95 ± 0.10	0
AR01 (13032-MU-A)	–	45.18 ± 5.08	11.47 ± 0.37	0

### Improvement of β-arbutin production via optimizing the fermentation process

**Fig. 3:**
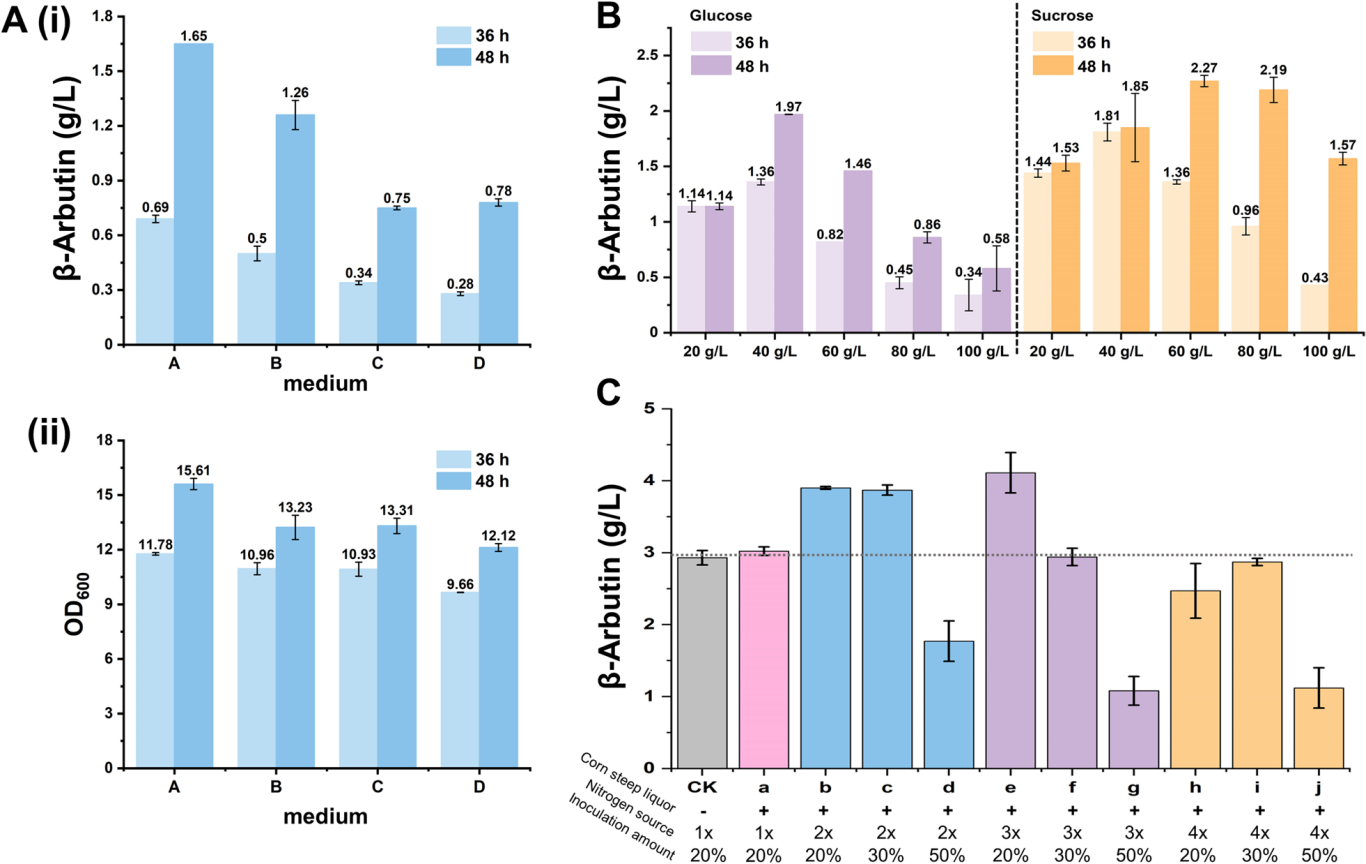
Optimization of the fermentation medium. **A** β-Arbutin production of AR01 cultivated in four fermentation media at 36 and 48 h (i). The OD_600_ of AR01 in four fermentation media at 36 h and 48 h (ii). **B** The β-arbutin production of AR01 after adding 20, 40, 60, 80, and 100 g/L glucose or sucrose to medium A at 36 and 48 h. **C** The β-arbutin production of AR01 at 20%, 30%, and 50% inoculation amount in medium A supplemented with 60 g/L sucrose, 5 g/L corn steep liquor, and 2-, 3-, and 4-times nitrogen sources

Optimizing the culture medium is crucial for producing specific target metabolites via microbial fermentation. Initially, strain AR01 produced only 45.18 mg/L β-arbutin after 72 h cultivation. Although the LB medium provides the necessary nutrients for cell growth, it may lack the essential components required for the β-arbutin biosynthetic pathway. Four candidate media were tested to determine the most suitable. Medium A was particularly conducive to β-arbutin accumulation, achieving a yield of 1.65 g/L at 48 h, exceeding the other media's yield (Fig. 3A). The optimal growth of strain AR01 in medium A suggests a theoretical correlation between strain growth and β-arbutin synthesis (Fig. 3A). Considering that the carbon source's type and quantity can affect the target product's yield, we conducted experiments with strain AR01 cultured in medium A supplemented with varying concentrations of glucose or sucrose (20, 40, 60, 80, and 100 g/L). The highest yield of β-arbutin was achieved in medium A containing 40 g/L sucrose, reaching 1.81 g/L at 36 h. As the fermentation progressed to 48 h, the yield of β-arbutin in medium A with 60 g/L sucrose increased to 2.27 g/L, representing a 1.38-fold enhancement compared to the initial β-arbutin yield in medium A (Fig. 3B). Therefore, the optimal sucrose concentration for β-arbutin production was 60 g/L. This finding is consistent with a previous study, where adding various glucose concentrations to *Y. lipolytica* significantly enhanced β-arbutin yield [25]. Subsequently, the inoculation amount and nitrogen source concentration were optimized in the fermentation medium of strain AR01. The maximum β-arbutin yield reached 4.11 g/L when utilizing 5 g/L corn steep liquor, three times more nitrogen source, and a 20% inoculum (Fig. 3C). Additionally, β-arbutin yields of 3.9 and 3.87 g/L were achieved with 20% and 30% inoculum, respectively, using 5 g/L corn steep liquor and two times more nitrogen source (Fig. 3C). Notably, these titers were higher than titer of the control group (2.93 g/L), suggesting that the biosynthesis of β-arbutin was significantly influenced by the quantity of the nitrogen source and the inoculation. Considering these factors, the optimal conditions for the subsequent fermentation test were determined to be medium A with two times more nitrogen sources, 5 g/L corn steep liquor, and 60 g/L sucrose with 20% inoculum.

### Engineering of the shikimate pathway in *C. glutamicum* to improve β-arbutin production

**Fig. 4:**
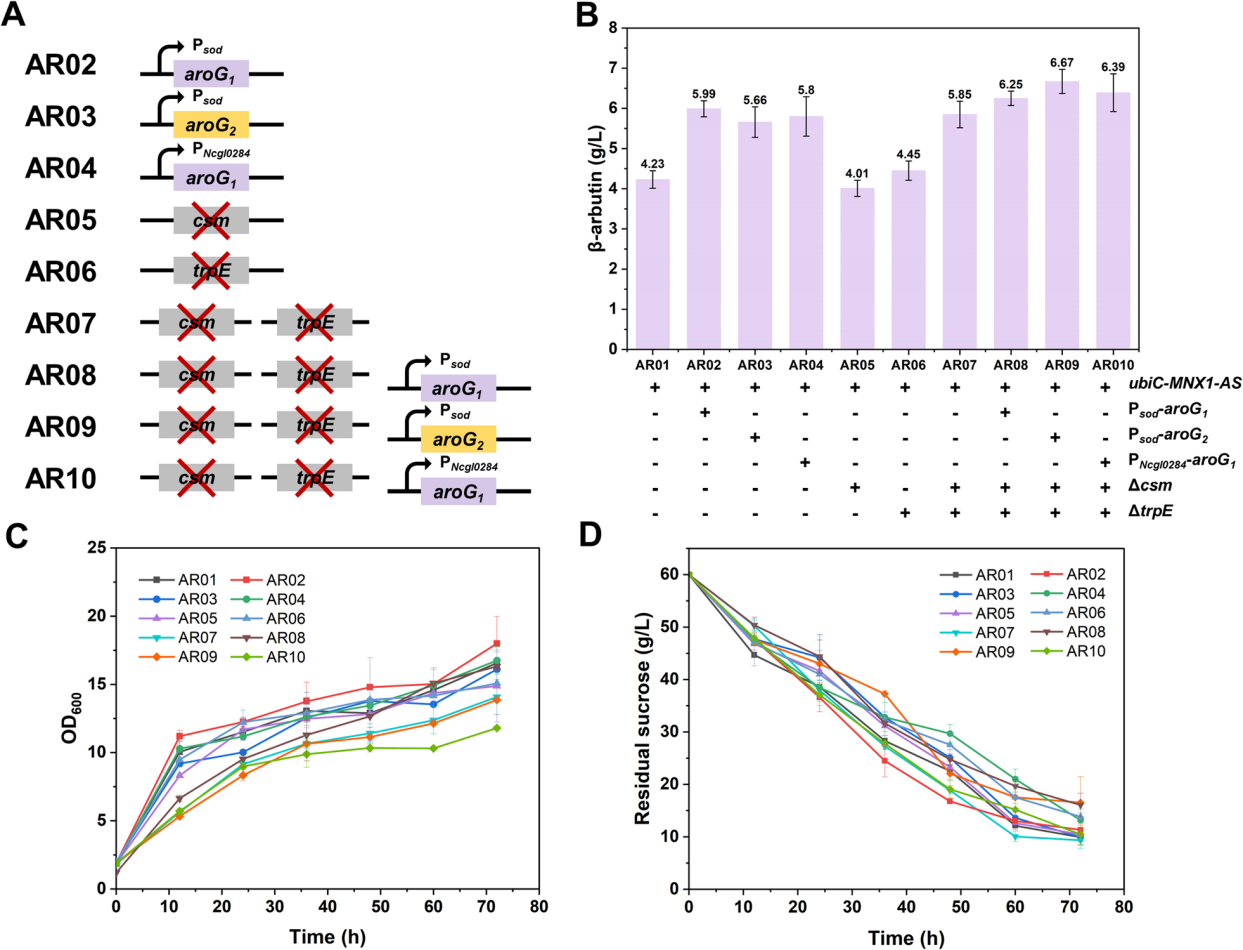
The effect of metabolic engineering of the shikimate pathway on β-arbutin production. **A** Genetic manipulation diagram of the engineered strains AR01 to AR10. **B** β-Arbutin production of each engineered strain at 72 h. **C** The growth curve of engineered strains. **D** The residual sucrose curve of engineered strains

To augment the carbon flux towards the β-arbutin synthesis pathway, modifications were made to the upstream shikimate pathway to enhance the supply of the precursor chorismate. 3-Deoxy-D-arabinoheptulosonate 7-phosphate synthase is a key enzyme in the shikimate pathway. In *C. glutamicum*, *aroG1* and *aroG2* encode the isozymes AroG1 and AroG2, respectively. The isozymes perform similar functions. Carbon flux through the shikimate pathway can be increased by overexpressing the endogenous *aroG* [34]. We replaced the original promoter of *aroG1* with two robust constitutive promoters, P_*Ncgl0284*_ and P_*sod*_, to generate engineered strains AR02 and AR04, respectively (Fig. 4A). Simultaneously, the original promoter of *aroG2* was substituted with a potent P_*sod*_ promoter, resulting in strain AR03. To assess the β-arbutin yield, these recombinant strains were used in a shake-flask fermentation experiment. HPLC analysis of the 72-h fermentation broth revealed that AR02, AR03, and AR04 produced 5.99, 5.66, and 5.80 g/L β-arbutin, respectively, which were 1.42, 1.34, and 1.37 times higher than the control, respectively (Fig. 4A). These results suggest that using the strong P_*Ncgl0284*_ and P_*sod*_ promoters to replace the original promoter of *aroG* effectively enhanced β-arbutin production. This finding is consistent with earlier reports indicating that the increased expression of *aroG* plays a crucial role in influencing the production of various metabolites in *C. glutamicum*, including chorismite [35], 2-phenylethanol [36], and shikimic acid [30].

Chorismate acts as a precursor of aromatic amino acids; thus, the aromatic amino acid synthesis pathway competes with that of β-arbutin. To redirect the metabolic flux towards the biosynthesis of β-arbutin, the genes *trpE* (encoding anthranilate synthase) and *csm* (encoding chorismate mutase) in the aromatic amino acid synthesis pathway were disrupted. This led to the construction of three recombinant strains: AR05 (AR01 with *csm* deletion), AR06 (AR01 with *trpE* deletion), and AR07 (AR01 with *csm* and *trpE* deletions) (Fig. 4A). The outcomes of the 72-h shake-flask fermentations revealed that AR05, AR06, and AR07 could accumulate 4.01, 4.45, and 5.85 g/L of β-arbutin, respectively (Fig. 4B). While individual inactivation of *csm* and *trpE* did not have a discernible effect on β-arbutin production, the simultaneous double deletion of these two genes resulted in a notable 38.5% increase in β-arbutin production compared to the control (4.11 g/L). Tryptophan was not detected in the fermentation broths of the strains AR05 and ARO7 (Table 2). Phenylalanine and tyrosine yields of strains AR06 and AR07 were significantly lower than those of the parent strain AR01 (Table 2). The decrease in the concentration of aromatic amino acids indicated that the deletion of *csm* and *trpE* effectively blocked competitive metabolic pathways. Natural nitrogen sources may have introduced residual amounts of phenylalanine and tyrosine into the fermentation medium. Therefore, as hypothesized, inactivating the competing metabolic pathway promoted the accumulation of β-arbutin. Additionally, under equivalent glucose consumption, the growth of strain AR07 exhibited a pronounced decline compared with that of the other strains (Fig. 4C, D). This observation suggests that the inactivation of the synthetic pathway for these amino acids exerts a distinct effect on cell growth. Based on these results, our hypothesis suggests that by disrupting competing metabolic pathways while simultaneously overexpressing *aroG1* and *aroG2*, the yield of β-arbutin can be further increased. To test this hypothesis, three recombinant strains, AR08, AR09, and AR10, were constructed by replacing the natural promoters of *aroG1* and *aroG2* with strong promoters in the AR07 strain (Fig. 4A). Although the growth rate of the strain remained lower than that of the starting strains, AR01, AR07, AR08, and AR09 exhibited further improvements in β-arbutin production, reaching 6.25, 6.67, and 6.39 g/L, respectively (Fig. 4B–D). The experimental results align with our hypothesis, indicating that enhancing *aroG* while simultaneously knocking out competing metabolic pathways can further promote the accumulation of β-arbutin.

**Table 2:** Analysis of by-products during fermentation process

Strains	β-Arbutin (g/L)	L-Try (mg/L)	L-Phe (mg/L)	L-Tyr (mg/L)
AR01	4.23 ± 0.22	147.28 ± 17.85	150.25 ± 11.26	450.65 ± 18.59
AR05	4.01 ± 0.20	0	136.78 ± 9.65	424.33 ± 22.87
AR06	4.45 ± 0.44	153.88 ± 14.68	55.98 ± 1.26	121.46 ± 15.28
AR07	5.85 ± 0.33	0	43.54 ± 0.89	88.95 ± 7.54
Medium	–	61.84 ± 3.26	65.27 ± 0.23	136.38 ± 4.96

### Increasing the supply of the UDP-glucose precursor for β-arbutin production

**Fig. 5:**
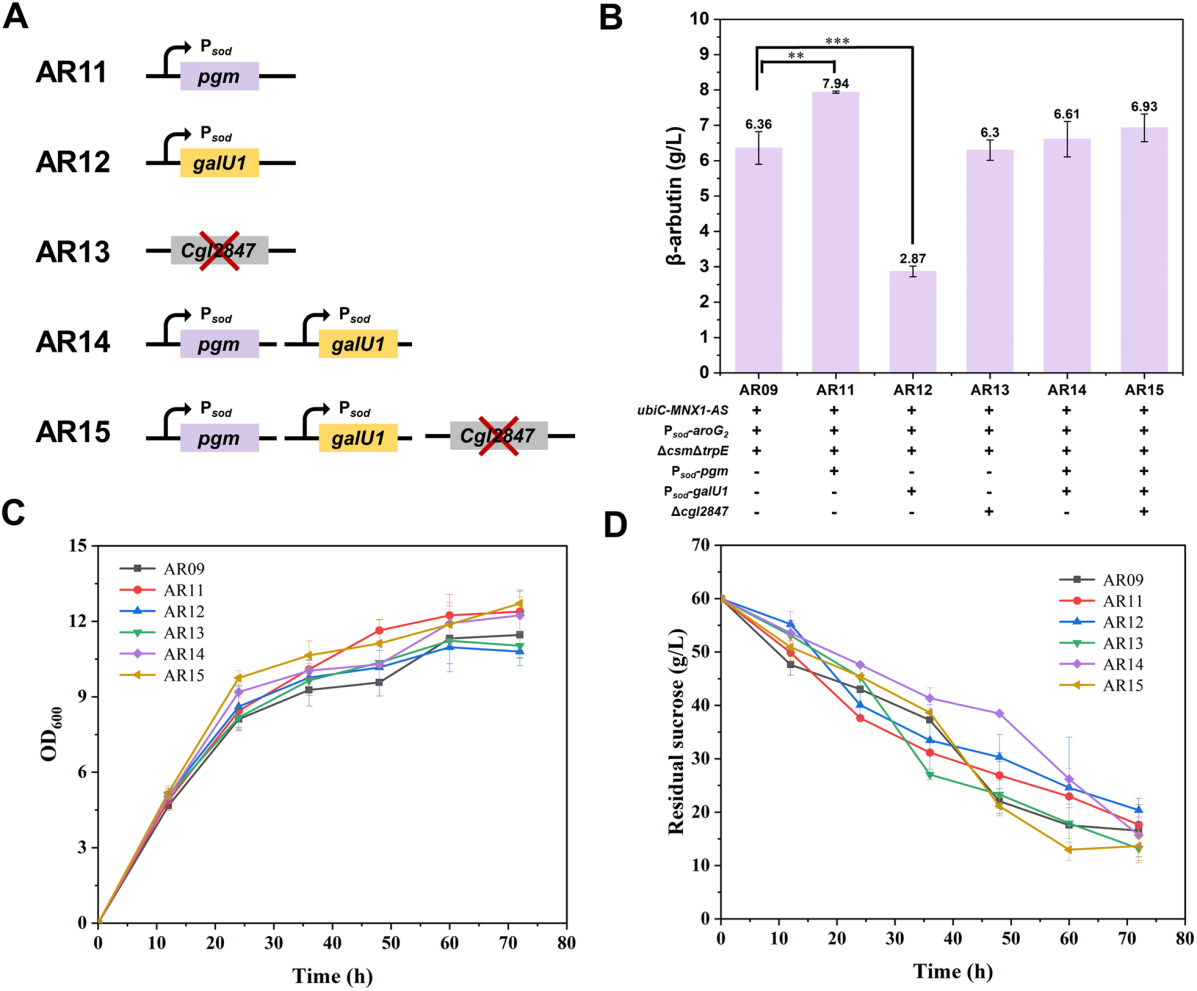
The effect of increasing UDPG availably on β-arbutin production. **A** Genetic manipulation diagram of engineered strains AR11 to AR15. **B** β-Arbutin production of engineered strains AR11 to AR15 at 72 h. **C** The growth curve of engineered strains AR11 to AR15. **D** The residual sucrose curve of engineered strains AR11 to AR15. **P < 0.05, ***P < 0.01

In the process of β-arbutin biosynthesis, the availability of UDP-glucose plays a crucial role in the glycosylation of HQ and is considered an essential co-substrate. Consequently, we hypothesized that the availability of UDP-glucose significantly influences β-arbutin production. General strategies to augment UDP-glucose availability involve amplifying genes associated with endogenous pathways and impeding pathways that consume UDP-glucose. For instance, previous studies have demonstrated that the overexpression of *pgm* (encoding phosphoglucomutase) and *galU1* (encoding UTP-glucose-1-phosphate uridylyltransferase) effectively enhanced the intracellular concentration of UDP-glucose [37]. We investigated the impact of elevated the availability of UDP-glucose supply on β-arbutin production. This involved replacing the inherent promoters of *pgm* and *galU1* with the potent promoter P_*sod*_ and incorporating a terminator region into the sequence of *Cgl2847* to diminish its expression. This resulted in the development of the engineered strains AR11 (AR09 with P_*sod*_-*pgm*), AR12 (AR09 with P_*sod*_-*galU1*), AR13 (AR09 with *Cgl2847* deletion), AR14 (AR09 with P_*sod*_-*pgm* and P_*sod*_-*galU1*), and AR15 (AR09 with P_*sod*_-*pgm*, P_*sod*_-*galU1*, and *Cgl2847* deletion) (Fig. 5A). The 72-h fermentation titer of β-arbutin of recombinant strains AR11, AR12, AR13, AR14, and AR15 reached 7.94, 2.87, 6.3, 6.61, and 6.93 g/L, respectively (Fig. 5B). The cell growth and glucose consumption rates were comparable between these strains (Fig. 5C, D). Compared to the parent strain AR09, only AR11 and AR15 demonstrated an increase in β-arbutin production titer. This observation implies that the overexpression of *pgm* was beneficial for the biosynthesis of β-arbutin, as illustrated in Fig. 5B. The intracellular UDP-glucose concentrations of AR11 and AR15 reached 544.67 ± 160.34 μg/g and 507.98 ± 23.71 μg/g, representing increases of 60% and 49.2%, respectively, compared to the control strain AR09 (340.44 μg/g) (Table 3). In contrast, overexpression of *galU1* alone in AR12 significantly decreased the production titer of β-arbutin, indicating the negative effect of *galU1* overexpression on the biosynthesis of β-arbutin. When *pgm* and *galU1* were overexpressed simultaneously, the adverse effects of *galU1* overexpression were partially mitigated. This phenomenon is likely attributable to UTP-glucose-1-phosphate uridylyltransferase, encoded by *galU1*, a bifunctional enzyme that catalyzes the interconversion between glucose-1-phosphate and UDP-glucose. Upon *galU1* overexpression, the direction of UDP-glucose conversion within the recombinant cells shifted towards glucose-1-phosphate. This shift reduced intracellular UDP-glucose concentrations to 280.40 ± 26.77 μg/g, which is lower than that of the control strain AR09, resulting in a significant reduction in β-arbutin production (Table 3). Additionally, the β-arbutin production titer of the recombinant strain AR15, which overexpressed *pgm* and *galU1* and weakened the expression of *Cgl2847*, was higher than that of AR09 but considerably lower than that of the recombinant strain AR11 (Fig. 5B). However, the yield and OD of the AR11 strain are among the highest, while its sugar consumption is relatively low. To investigate this further, we measured the intracellular byproduct concentrations in strains AR09 and AR11. As shown in Table 4, the accumulation of intermediate metabolites in AR11 is lower compared to AR09. Specifically, the intracellular concentrations of citrate, aconitate, isocitrate, fructose-1,6-bisphosphate, 2-phosphoglycerate, phosphoenolpyruvate, L-lactate, and α-ketoglutarate in AR11 were significantly lower than those in AR09. This phenomenon may be linked to the intracellular accumulation of glucose-1-phosphate, which promotes sugar metabolism.

**Table 3:** Analysis of intracellular UDP-glucose of the engineering strains

Strains	AR09	AR11	AR12	AR13	AR14	AR15
UDP-glucose (μg/g DCW)	340.44 ± 78.48	544.67 ± 160.34	280.40 ± 26.77	331.58 ± 32.54	485.12 ± 45.68	507.98 ± 23.71
Glucose-1-phosphate (μg/g DCW)	156.12 ± 37.14	265.73 ± 7.63	286.68 ± 45.36	128.64 ± 22.14	223.12 ± 34.56	208.57 ± 35.68

**Table 4:** Analysis of intracellular by-products of strain AR09 and AR11

Strains/products	AR09 (μg/g DCW)	AR11 (μg/g DCW)
Citrate	18.62 ± 1.78	7.13 ± 1.05
Aconitate	0.74 ± 0.09	0.38 ± 0.06
Isocitrate	0.36 ± 0.00	0.12 ± 0.02
Succinate	288.14 ± 6.50	242.47 ± 25.16
Malate	67.86 ± 1.22	60.00 ± 4.85
Fructose-1,6-diphosphate	942.64 ± 105.82	295.00 ± 11.34
2,3-Diphosphoglycerate	0.36 ± 0.05	0.27 ± 0.01
2-Phosphoglycerate	27.64 ± 2.35	10.38 ± 1.69
Phosphoenolpyruvate	34.98 ± 0.93	11.04 ± 1.39
6-Phosphogluconate	0.77 ± 0.09	0.36 ± 0.02
Sedoheptulose-7-phosphate	67.61 ± 0.61	69.48 ± 6.00
3-Phosphoglycerate	200.41 ± 30.60	47.40 ± 6.61
L-Lactate	4903.89 ± 668.65	12,032.34 ± 904.93
Pyruvate	46.40 ± 1.09	28.48 ± 2.91
Glucose-1-phosphate	156.12 ± 37.14	265.73 ± 7.63
Erythritose-4-phosphate	41.95 ± 8.03	45.88 ± 1.30
α-Ketoglutarate	182.05 ± 16.52	35.59 ± 5.62
Oxaloacetate	446.61 ± 58.07	1029.42 ± 31.15

## Conclusions

In this study, we have achieved the heterologous expression of *ubiC*, *MNX1*, *AS*, and the assimilation of the β-arbutin biosynthesis pathway in *C. glutamicum*. Following optimization of the nitrogen source, carbon source, and medium inoculum, the production titer of β-arbutin was further improved. Additionally, by enhancing *aroG* expression, eliminating competing metabolic pathways, and optimizing UDP-glucose synthesis, we obtained the final engineered strain, AR11. After 72 h of fermentation, AR11 demonstrated a β-arbutin yield of 7.94 g/L. This study is the first to report β-arbutin biosynthesis by *C. glutamicum*.

## Materials and methods

### Strains, plasmids, and culture conditions

*E. coli* DH5α (TOLOBIO, Shanghai, China) was used for the cloning and propagating of recombinant plasmids. *C. glutamicum* ATCC13032 served as the host for β-arbutin production. Table 5 provides a comprehensive list of all the plasmids and strains used in this study. Recombinant *C. glutamicum* strains were inoculated into a seed medium contained 50 g/L glucose, 10 g/L yeast extract, 30 g/L corn steep liquor (Aladdin, China), 0.25 g/L MgSO_4_, 4 g/L (NH_4_)_2_SO_4_, 1 g/L KH_2_PO_4_, 1 g/L K_2_HPO_4_·3H_2_O, 0.01 g/L FeSO_4_·7H_2_O, and 0.02 g/L MnSO_4_·H_2_O, and were subsequently cultured at 220 rpm and 32 ℃ for 12 h. Fermentation medium A used for the production of β-arbutin by *C. glutamicum* contained 80 g/L sucrose, 5 g/L yeast extract, 1.5 g/L urea, 2 g/L peptone, 0.5 g/L KH_2_PO_4_, 0.5 g/L K_2_HPO_4_·3H_2_O, 0.02 g/L FeSO_4_·7H_2_O, 0.02 g/L MnSO_4_·H_2_O, 0.5 mg/L biotin, and 1 mg/L thiamine. Medium B contained medium A supplemented with 5 g/L citric acid. Medium C comprised medium A, containing 80 g/L glucose instead of sucrose. Medium D comprised medium C supplemented with 5 g/L citric acid.

**Table 5:** Plasmids and strains used in this study

Plasmid/strains	Description	Source
Plasmid		
pXMJ19	A shuttle expression vector, Chl^R^	Lab stock
pEC-XK99E	A shuttle expression vector, Km^R^	Lab stock
pK18*mobsacB*	Mobilizable vector, allows for selection of double crossover in *C. glutamicum*, Km^R^, *sacB*	Lab stock
pXMJ19-A	A derivative of pXMJ19, harboring codon-optimized *AS* gene from *Rauvolfia serpentina* under its native promoter	This study
pXMJ19-M	A derivative of pXMJ19, harboring codon-optimized *MNX1* gene from *Candida parapsilosis* CBS604 under its native promoter	This study
pXMJ19-U	A derivative of pXMJ19, harboring codon-optimized *ubiC* gene from *E. coli* under its native promoter	This study
pXMJ19-AM	A derivative of pXMJ19, harboring codon-optimized *AS-MNX1* gene under its native promoter	This study
pXMJ19-MU	A derivative of pXMJ19, harboring codon-optimized *MNX1-ubiC* gene under its native promoter	This study
pXMJ19-AMU	A derivative of pXMJ19, harboring codon-optimized *AS-MNX1- ubiC* gene under its native promoter	This study
pXMJ19-MUA	A derivative of pXMJ19, harboring codon-optimized *MNX1-ubiC*-*AS* gene under its native promoter	This study
PEC-XK99E-A	A derivative of pEC-XK99E, harboring codon-optimized *AS* gene under its native promoter	This study
pK18-P_*sod*_-*aroG*_*1*_	A derivative of pK18*mobsacB*, harboring P_*sod*_-*aroG*_*1*_ fragment	This study
pK18-P_*sod*_-*aroG*_*2*_	A derivative of pK18*mobsacB*, harboring P_*sod*_-*aroG*_*2*_ fragment	This study
pK18-P_*Ncgl0284*_-*aroG*_*1*_	A derivative of pK18*mobsacB*, harboring P_*Ncgl0284*_-*aroG*_*1*_ fragment	This study
pK18-Δ*csm*	A derivative of pK18*mobsacB*, harboring Δ*csm* fragment	This study
pK18-Δ*trpE*	A derivative of pK18*mobsacB*, harboring Δ*trpE* fragment	This study
pK18-P_*sod*_-*pgm*	A derivative of pK18*mobsacB*, harboring P_*sod*_-*pgm* fragment	This study
pK18-P_*sod*_-*galU1*	A derivative of pK18*mobsacB*, harboring P_*sod*_-*galU1* fragment	This study
pK18-Δ*cgl2847*	A derivative of pK18*mobsacB*, harboring Δ*cgl2847*fragment	This study
Strains		
*E. coli* DH5α	Gene cloning host strain	Transgen
*C. glutamicum* ATCC 13032	Type strain	ATCC
13032-CK	*C. glutamicum* ATCC 13032 with pXMJ19	This study
13032-A	*C. glutamicum* ATCC 13032 with pXMJ19-A	This study
13032-M	*C. glutamicum* ATCC 13032 with pXMJ19-M	This study
13032-U	*C. glutamicum* ATCC 13032 with pXMJ19-U	This study
13032-AM	*C. glutamicum* ATCC 13032 with pXMJ19-AM	This study
13032-MU	*C. glutamicum* ATCC 13032 with pXMJ19-MU	This study
13032-AMU	*C. glutamicum* ATCC 13032 with pXMJ19-AMU	This study
13032-MUA	*C. glutamicum* ATCC 13032 with pXMJ19-MUA	This study
AR01 (13032-MU-A)	*C. glutamicum* ATCC 13032 with pXMJ19-MU and pEC-XK99E-A	This study
AR02	AR01 with P_*sod*_ promoter inserted in the upstream of *aroG*_*1*_	This study
AR03	AR01 with P_*sod*_ promoter inserted in the upstream of *aroG*_*2*_	This study
AR04	AR01 with P_*Ncgl0284*_ promoter inserted in the upstream of *aroG*_*1*_	This study
AR05	AR01 with *csm* deletion	This study
AR06	AR01 with *trpE* deletion	This study
AR07	AR01 with *csm* and *trpE* deletion	This study
AR08	AR07 with P_*sod*_ promoter inserted in the upstream of *aroG*_*1*_	This study
AR09	AR07 with P_*sod*_ promoter inserted in the upstream of *aroG*_*2*_	This study
AR10	AR07 with P_*Ncgl0284*_ promoter inserted in the upstream of *aroG*_*1*_	This study
AR11	AR09 with P_*sod*_ promoter inserted in the upstream of *pgm*	This study
AR12	AR09 with P_*sod*_ promoter inserted in the upstream of *galU1*	This study
AR13	AR09 with *cgl2847* deletion	This study
AR14	AR11 with P_*sod*_ promoter inserted in the upstream of *galU1*	This study
AR15	AR14 with *cgl2847* deletion	This study

### Construction of recombinant strains

All primers were synthesized using GenScript (Nanjing, China). The sequences are provided in the Supplementary Materials (Table S1). The *ubiC*, *MNX1*, and *AS* genes used for β-arbutin synthesis were synthesized by Ruimian Biotech (Shanghai, China). These genes were amplified using the polymerase chain reaction. The resulting products were purified and cloned into the plasmids pXMJ19 and pEC-XK99E for gene overexpression. Deletion of *trpE*, *csm*, and *cgl2847* was performed in *C. glutamicum* as previously described [38, 39]. The *sod* promoter and *ncgl0284* promoter were used for the chromosome-based overexpression of *aroG*_*1*_, *aroG*_*2*_, *pgm*, and *galU1*. The recombinant plasmids were transformed into *C. glutamicum* by electroporation, as previously described [38, 39].

### Growth inhibition of *C. glutamicum* in response to β-arbutin and HQ treatment

*C. glutamicum* ATCC13032 HQ and β-arbutin tolerance were assayed in liquid LB medium. After adding β-arbutin (final concentrations 5–80 g/L) or HQ (final concentrations 0.5–10 g/L), cell growth was examined by sampling every 2 h. To verify whether the three exogenous genes (*ubiC*, *MNX1*, and *AS*) were expressed in *C. glutamicum*, fermentation was performed in a liquid LB medium or LB medium with pHBA or HQ as a substrate (final concentration 1 g/L).

### Analysis of extracellular metabolites

Samples were collected every 12 h to measure β-arbutin production, sucrose consumption, and the optical density at 600 nm (OD_600_). The fermentation broths were analyzed by HPLC at 48 and 72 h to determine the presence of the corresponding products. The concentrations of pHBA, HQ, β-arbutin, and aromatic amino acids were quantitatively analyzed by HPLC using a model 2847 apparatus with a Symmetry C18 column (5 μm, 4.6 × 250 mm; Waters, USA) and an ultraviolet–visible detector. pHBA was separated through isocratic elution using 20% methanol–water containing 5% acetic acid as the eluant at a flow rate of 0.8 mL/min with a column temperature of 30 °C and a detection wavelength of 256 nm. HQ and β-arbutin were separated using 15% methanol–water isocratic elution at a 1 mL/min flow rate at room temperature and a detection wavelength of 280 nm. Aromatic amino acids were separated using 6% acetonitrile–water at a 1 mL/min flow rate at 30 °C and a detection wavelength of 215 nm. The concentrations of glucose and sucrose were quantified by HPLC using a model e2695 device (Waters) with a Carbohydrate ES column (5 μm, 4.6 × 250 mm) and a model 2414 refractive index detector (Waters).

### Analysis of intracellular metabolites

Each individual sample was collected at 24 h in a 2 mL centrifuge tube. Subsequently, 1000 μL of extract solvent (methanol–water, 3:1, pre-cooled at − 20 °C) was added to the samples, followed by vortexing for 30 s, incubation at − 20 °C for one hour, and centrifugation. The clear supernatant was then subjected to LC–MS/MS analysis. UHPLC separation was performed using a Waters ACQUITY H-class plus UPLC System equipped with a Waters ACQUITY UPLC BEH C18 column (2.1 × 100 mm, 1.7 μm). Mobile phase A consisted of 0.1% formic acid in water, while mobile phase B comprised acetonitrile. The flow rate was set at 300 μL/min, and the column temperature was maintained at 40 °C. The auto-sampler temperature was set at 10 °C, with an injection volume of 1 μL. Assay development utilized a Waters Xevo TQ-XS triple quadrupole mass spectrometer equipped with an electrospray ionization (ESI) interface. Typical ion source parameters included a capillary voltage of -2500 V, a Cone voltage of 30 V, a desolvation temperature of 550 °C, a desolvation gas flow of 1000 L/h, a collision gas flow of 0.15 mL/min, and a nebulizer gas flow of 7 Bar. MRM parameters for each targeted analyte were optimized by directly injecting standard solutions of individual analytes into the API source of the mass spectrometer. At least two MRM transitions (Q1/Q3 pairs) were obtained per analyte, with the two most sensitive transitions used in the MRM scan mode to optimize collision energy for each Q1/Q3 pair. The most sensitive and selective Q1/Q3 pairs were selected for quantitative monitoring, while additional transitions served as qualifiers to verify the identity of target analytes. Data acquisition and processing were performed using Waters MassLynx V4.2 Workstation Software.

### Supplementary Information

Supplementary Material 1.Table S1: Primers used in this study; Table S2. Codon optimized sequences of *ubiC*, *MNX1*, and *AS* for *C. glutamicum*.

## Data Availability

Not applicable.

## Abbreviations

LB: Luria–Bertani
HQ: Hydroquinone
GTs: Glycosyltransferases
pHBA: P-Hydroxybenzoic acid
FAD: Flavin adenine dinucleotide
MNX1: Enzyme 4-hydroxybenzoate 1-hydroxylase
AS: β-Arbutin synthase
GRAS: Generally recognized as safe
UDP: Uridine diphosphate
PCR: Polymerase chain reaction
HPLC: High-performance liquid chromatography
DAHP: 3-Deoxy-D-arabinoheptulosonate 7-phosphate
