# Supplementary material for: De novo biosynthesis of β-arbutin in Corynebacterium glutamicum via pathway engineering and process optimization
Source: Biotechnol Biofuels Bioprod. 2024 Jun 25;17:88. doi: 10.1186/s13068-024-02540-2 (PMC11197339; doi:10.1186/s13068-024-02540-2)
Supplement: Supplementary file 1 — Supplementary Material 1.Table S1: Primers used in this study; Table S2. Codon optimized sequences of ubiC, MNX1, and AS for C. glutamicum. [file 13068_2024_2540_MOESM1_ESM.docx]

**Supporting Information**

**Table S1. Primers used in this study.**

| Primer name | Sequence (5’-3’) |
| --- | --- |
| AMU-F | ctgcaggtcgactctaga*GGATCC*GAAAGGATTTTTTACCCATGGAAC |
| AMU-R | cgccaaaacagccaagct*GAATTC*TTAGTACAGTGGGGAAGCTG |
| *AS*-R | cgccaaaacagccaagct*GAATTC*GTGCCTGGACAGCCATGTATATC |
| *MNX1*-F | ctgcaggtcgactctaga*GGATCC*CAAGGAGATATACATGGCTGTCC |
| *MNX1*-R | cgccaaaacagccaagct*GAATTC*AGCTCCTTGGTTAGCCGGAAG |
| *UbiC*-F | ctgcaggtcgactctaga*GGATCC*CCAAGGAGCTTTAAAACAGCAT |
| pXMJ19-F | GCACTCCCGTTCTGGATAATG |
| pXMJ19-R | GTCTTTCGACTGAGCCTTTCG |
| pXMJ19-MUA-F | gcttccccactgtactaa*GAATTC*GAAAGGATTTTTTACCCATGGAAC |
| pXMJ19-MUA-R | cgccaaaacagccaagct*GAATTC*CGTGCTTGGACAGCCATGTATAT |
| P*_tac_*-A-F | acttggtggtttcgc*TCTAGA*CGGCTCGTATAATGTGTGGAAT |
| P*_tac_*-A-R | acggctggaacagatgaa*AAGCTT*CGTGCTTGGACAGCCATGTATAT |
| pEC-XK99E-A-F | cacaggaaacagaccatg*GAATTC*GAAAGGATTTTTTACCCATGGAAC |
| pEC-XK99E-A-R | ctgcaggtcgactctaga*GGATCC*CGTGCTTGGACAGCCATGTATAT |
| *csm*-up-F | aacgacggccagtgccaagctCGGTGTACAAGTTGAGGACTACC |
| *csm*-up-R | gtggcagaatagtgtgcatgactaACTCGGATAAGTTATCCACAGGTAG |
| *csm*-down-F | TAGTCATGCACACTATTCTGCCAC |
| *csm*-down-R | cggtacccggggatcctctagTTGCCGACGAGCGTAGAAATC |
| *trpE*-up-F | aacgacggccagtgccaagctTGTCAGGATCGTGCAGTGAAG |
| *trpE*-up-R | attagttcgcgagaagctgttcgGGCACCTACCGAGGAAATCAT |
| *trpE*-down-F | CGAACAGCTTCTCGCGAACTAAT |
| *trpE*-down-R | cggtacccggggatcctctagCTACGAGATCCTCAAGGGTGTAGC |
| *trpE*-check-F | TTTCACCCTGAGTCAGTGCTAAG |
| P*_sod_*-F | TGCCAATTATTCCGGGCTTG |
| P*_sod_*-R | TCCGCACCGAGCATATACATCTT |
| P*_NCgl0284_*-F | AACTGTGCCACTAATACGGATAG |
| P*_NCgl0284_*-R | CAATTTCGCCTGCTTCCGATT |
| *aroG_1_*-up-F | aacgacggccagtgccaagctCTTGTGAGCGCTTCTTTGATC |
| *aroG_1_*-up-R(P*_sod_*) | caagcccggaataattggcaatGGGATGGGGTGAATTTAGG |
| *aroG_1_*-up-R(P*_NCgl0284_*) | ctatccgtattagtggcacagttatGGGATGGGGTGAATTTAGG |
| *aroG_1_*-down-F(P*_sod_*) | aagatgtatatgctcggtgcggatGCATAGCCCTGAAAGGCAAG |
| *aroG_1_*-down-F(P*_NCgl0284_*) | aatcggaagcaggcgaaattgTGCATAGCCCTGAAAGGCAAG |
| *aroG_1_*-down-R | cggtacccggggatcctctagTCGTCGGAGGTTCCGAAGAAG |
| *aroG_2_*-up-F | aacgacggccagtgccaagctCAGTGCTTTTCTCAGCGTCTG |
| *aroG_2_*-up-R | caagcccggaataattggcaTCATAGCACGATATCGGTGTT |
| *aroG_2_*-down-F | aagatgtatatgctcggtgcggaATGAATAGGGGTGTGAGTTGG |
| *aroG_2_*-down-R | cggtacccggggatcctctagGCTCGTAATCCACCAGCAAAG |
| *pgm*-up-F | aacgacggccagtgccaagctGGCTTCAACTGGGGAAATTGTT |
| *pgm*-up-R | caagcccggaataattggcaCTTAAAACACCAATACTTTCTCTTCATCTTCCCCT |
| *pgm*-down-F | aagatgtatatgctcggtgcggaGTTTTAAGGAGCAAACATGGCACATG |
| *pgm*-down-R | cggtacccggggatcctctagGGATGGCATCGATGTTGACCACGT |
| *galU1*-up-F | aacgacggccagtgccaagctCCTTGGTGCCAGCAGGAATT |
| *galU1*-up-R | caagcccggaataattggcaCGCAACCCCAATCGTCGTAC |
| *galU1*-down-F | aagatgtatatgctcggtgcggaGGCAGGTACTCTTGGTTCCATG |
| *galU1*-down-R | cggtacccggggatcctctagAAGCAAATCAATCGCGTCGGTGAG |
| T-check-F | GGCGGTCAGATGATCGCCCTT |
| *cgl2847*-up-F | aacgacggccagtgccaagctCGTTGGAGTTGTCCAATGTGGCT |
| *cgl2847*-up-R | atcatctgaccgcccaagcttgggttcgtctagaACTCCGGATTCCACGCGATCT |
| *cgl2847*-down-F | **CAAGCTTGGGCGGTCAGATGATCGCCCTTTTTTTTTT**GCGGTCAAAGACACCATCACTC |
| *cgl2847*-down-R | cggtacccggggatcctctagTCGCGGAATTCTTGCCATTCAGTG |

Note: The lower-case sequence is the homologous arms, the italic underline sequence is the enzyme digestion site, and the coarse underline is the terminator sequence.

**Table S2. Codon optimized sequences of *ubiC*, *MNX1* and *AS* for *C. glutamicum.***

| Codon optimized sequences of *ubiC* |
| --- |
| ccaaggagctttaaaacagcATGTCCCACCCAGCACTGACCCAGCTGCGCGCACTGCGTTACTGCAAGGAGATCCCAGCACTGGACCCTCAGCTGCTGGACTGGCTGCTGCTGGAAGACTCCATGACCAAGCGCTTCGAGCAGCAGGGCAAGACCGTCTCCGTGACCATGATCCGCGAGGGCTTCGTCGAGCAGAACGAGATCCCAGAAGAACTGCCACTGCTGCCAAAGGAGTCCCGCTACTGGCTGCGCGAGATCCTGCTGTGCGCTGATGGCGAGCCTTGGCTGGCAGGCCGTACCGTTGTCCCAGTGTCCACCCTGTCCGGCCCAGAGCTGGCTCTGCAGAAGCTGGGCAAGACCCCACTGGGCCGCTACCTGTTCACCTCCTCCACCCTGACCCGCGACTTCATCGAAATCGGCCGCGACGCAGGCCTGTGGGGTCGTCGTTCTCGCCTGCGTCTGTCCGGCAAGCCACTGCTGCTGACCGAGCTGTTCCTGCCAGCTTCCCCACTGTACTAA |
| Codon optimized sequences of *MNX1* |
| caaggagatatacATGGCTGTCCAGGCACCATCCAAGACCTACGGCTTCCAGAAGGCTCCAATCCAGCTGACCTTCGTGGTCGTCGGCGCTGGCCTGGGTGGTGTTGCTGCTTCCATCTGCCTGCGCCTGGCAGGCCACCGTGTGATCCTGCTGGAAGCTGCAACCGAACTGGGCGAGGTCGGCGCTGGTATCCAGATCCCACCACCATCCACCAAGATCCTGAAGGCAATCGGCGTCCTGGACGCTGTCGATAAGGTGTCCATCCACCCACACGACATCCTGGTCAAGAAGTACAAGGGCGAACTGCTGTCCACCCAGAACCTGGTGCCATACGTCCTGGAGAAGTACGATGGCATGTACCTGCACATCCACCGCGCAGATTACCACAAGGTCCTGGTCGACCGCGCAGAGGAACTGGGCGTCGAGATCCACACCAACTCCCGCGTGGTGGACATCGATTTCGAAAAGGCAACCGTGACCACCGCTACCGGCAAGCAGTACTCCGGCGACGTGATCGTCGGCTACGATGGCGTGCGCTCCCAGACCCGTGCTCTGCTTACCGGCGATTCCTCCGGCGCATACGATACCGGCGACCTGGCTTACCGCGCTCTGATCAAGGTCGAAGACATGAAGAAGGTGCCAGGCCTGGAGAAGTTCTACGCTAACCCAAACATCAACTTCTGGTGGGGCCCAACCATGCACATCGTCATGTACTTCCTGCACGAAGGCGAGATCTGCAACGTGGTCGCTCTGTGCCCAGACACCCTGCCAAAGGGCGTCCTGAAGCAGGATGCTTCCCAGGAAGAACTGCTGGATCTGGTCAAGGGCTGGGATCAGGACCTGACCACCGTCTTCAAGCTGATCACCTCCGTCTCCAAGTGGCGCCTGCAGGACTCCCGCGAACTGAAGACCTGGGTGAACTCCAAGACCGGCAACTTCATCATCCTGGGCGATGCATCCCACTCCACCCTGCCATACCTGGCATCCGGCGCATCCCAGGCTGTGGAGGATGGCGCAGTCCTGGCAGGCCTGTTCTCCAAGATCGAACTGCGCGACCAGATCCCACAGCTGCTGCAGATGACCGAAAACCTGCGCAAGTGGCGCTCCTCCCAGGTGGTGCGTGGCTCTCACCAGTGCCAGGATATTTACCACCTGCCAGATGGCGAGCTGCAGGAGATCCGCGATTCCTACCTGTACGACAAGCAGCCAGAGCTGGGCTGCCCAAACCGCTTCGCTGACCCAGTGTTCCAGGACTTCCTGTGGGGCTACAACGCATTCGACGAGGTCGAACGCGCTTGGAAGGAGTTCAAGGCTGGCGGCAACCCAACCTACACCTACCCAAACCTGTACAAGCCAAAGTCCTCCGGCGAGAAGGATGTGTCCGGCGGCGGTGCTGCAGCAACCCTTGCAGCAGGCAACACCCCAGCAGCTCCACTGTCCGCTTCCGGCTAA |
| Codon optimized sequences of *AS* |
| gaaaggattttttacccATGGAACACACCCCACACATCGCTATGGTGCCAACCCCAGGCATGGGCCACCTGATCCCACTGGTCGAATTTGCAAAGCGCCTGGTGCTGCGCCACAACTTCGGCGTCACCTTCATCATCCCAACCGATGGCCCACTGCCAAAGGCACAGAAGTCCTTCCTGGACGCTCTGCCAGCTGGCGTGAACTACGTCCTGCTGCCACCAGTGTCCTTCGATGACCTGCCAGCAGATGTCCGCATCGAGACCCGCATCTGCCTGACCATCACCCGCTCCCTGCCATTCGTGCGCGATGCAGTGAAGACCCTGCTGGCAACCACCAAGCTGGCAGCTCTGGTCGTGGATCTGTTCGGCACCGACGCTTTCGATGTGGCAATCGAGTTCAAGGTCTCCCCATACATCTTCTACCCAACCACCGCAATGTGCCTGTCCCTGTTCTTCCACCTGCCAAAGCTGGATCAGATGGTCTCCTGCGAGTACCGCGATGTCCCAGAGCCACTGCAGATCCCAGGCTGCATCCCAATCCACGGCAAGGACTTCCTGGACCCTGCACAGGACCGCAAGAACGACGCATACAAGTGCCTGCTGCACCAGGCTAAGCGCTACCGCCTGGCAGAGGGCATCATGGTGAACACCTTCAACGACCTGGAGCCAGGCCCACTGAAGGCACTGCAGGAAGAAGACCAGGGCAAGCCACCAGTCTACCCAATCGGCCCACTGATCCGCGCAGATTCCTCCTCCAAGGTCGATGATTGCGAATGCCTGAAGTGGCTGGATGACCAGCCACGCGGCTCCGTCCTGTTCATCTCCTTCGGCTCCGGCGGCGCAGTCTCCCATAACCAGTTCATCGAGCTGGCACTGGGCCTGGAAATGTCCGAGCAGCGCTTCCTGTGGGTCGTGCGCTCCCCAAACGACAAGATCGCTAACGCTACCTACTTCTCCATCCAGAACCAGAACGATGCACTGGCATACCTGCCAGAGGGCTTCCTGGAGCGCACCAAGGGCCGTTGCCTGCTGGTCCCATCCTGGGCTCCACAGACCGAAATCCTGTCCCACGGCTCCACCGGCGGCTTCCTTACCCACTGCGGCTGGAACTCCATCCTGGAGTCCGTGGTGAACGGCGTCCCACTGATCGCATGGCCACTGTACGCAGAGCAGAAGATGAACGCAGTGATGCTGACCGAGGGCCTGAAGGTCGCTCTGCGCCCAAAGGCAGGCGAAAACGGCCTGATCGGCCGCGTCGAGATCGCAAACGCTGTGAAGGGCCTGATGGAAGGCGAGGAAGGCAAGAAGTTCCGCTCCACCATGAAGGATCTGAAGGATGCTGCATCCCGCGCACTGTCCGATGACGGCTCCTCCACCAAGGCTCTGGCTGAGCTGGCTTGCAAGTGGGAGAACAAGATCTCCTCCACCTAA |
